# Visual Electrodiagnostic Testing in Birdshot Chorioretinopathy

**DOI:** 10.1155/2015/680215

**Published:** 2015-07-13

**Authors:** Radouil Tzekov, Brian Madow

**Affiliations:** Department of Ophthalmology, Morsani College of Medicine, University of South Florida, 12901 Bruce B. Downs Boulevard, P.O. Box MDC 21, Tampa, FL 33612, USA

## Abstract

Birdshot chorioretinopathy (BSCR) is a rare form of autoimmune posterior uveitis that can affect the visual function and, if left untreated, can lead to sight-threatening complications and loss of central vision. We performed a systematic search of the literature focused on visual electrophysiology studies, including electroretinography (ERG), electrooculography (EOG), and visual evoked potentials (VEP), used to monitor the progression of BSCR and estimate treatment efficacy. Many reports were identified, including using a variety of methodologies and patient populations, which makes a direct comparison of the results difficult, especially with some of the earlier studies using nonstandardized methodology. Several different electrophysiological parameters, like EOG Arden's ratio and the multifocal ERG response densities, are reported to be widely affected. However, informal consensus emerged in the past decade that the full-field ERG light-adapted 30 Hz flicker peak time is one of the most sensitive electrophysiological parameters. As such, it has been used widely in clinical trials to evaluate drug safety and efficacy and to guide therapeutic decisions in clinical practice. Despite its wide use, a well-designed longitudinal multicenter study to systematically evaluate and compare different electrophysiological methods or parameters in BSCR is still lacking but would benefit both diagnostic and therapeutic decisions.

## 1. Introduction

Birdshot chorioretinopathy (BSCR) (ICD-9 363.20) is a rare form of slowly progressive, potentially blinding, bilateral posterior uveitis, likely of autoimmune origin, in which multiple hypopigmented choroidal lesions are scattered throughout the posterior pole. The term “birdshot retinopathy” was officially proposed by Ryan and Maumenee in 1980 based on the characteristic fundus appearance [1], although it is likely that description of the same condition was available before that, probably as early as 1949 [2]. The presence of the disease is strongly associated with the most prevalent subtypes of the human leucocyte antigen- (HLA-) A^*∗*^29 in Caucasians [3]; however, the role of this antigen in the pathophysiology of the disease is uncertain and, therefore, its presence is not essential for the establishment of BSCR diagnosis [4]. The disease is relatively uncommon, with an estimated occurrence in ~3% of all uveitis patients [5] and 6–8% of patients with posterior uveitis [2], affecting slightly more females (54–58%) than males in their 50s or 60s [2, 6]. It can include a variety of ocular complications, with the most frequent ones being macular edema in 50% and optic disc edema in 24% of the patients [2]. In more than 90% of the cases, both eyes are symmetrically involved and the densest concentration of lesions is around the optic disc, while few can be seen in the macula. There is also a tendency for the lesions to progress into large areas of choroidal depigmentation and in 61% of the cases narrowing of the retinal arteries is noted [7]. In addition, retinal vasculitis is often present, primary as phlebitis in the posterior pole [8] often detected as a vascular leakage on fluorescein angiography [7].

The pathogenesis of the disease is still poorly understood. Only two histological reports have been published indicating that the hypopigmented choroidal lesions represent nodules of lymphocyte aggregation, a sign of nongranulomatous nodular infiltration of the choroid [9, 10]. The application of enhanced depth imaging in spectral-domain optic coherence tomography (SD-OCT) showed recently significant choroidal thinning and disruption of the outer retinal substructures in extramacular locations [11].

Treatment of BSCR involves oral and periocular corticosteroids, which can be effective in the short-term and during the early stages of the disease. Additionally, various immunosuppressants, such as cyclosporine, azathioprine, and cyclophosphamide, have been tried as long-term therapy with mixed results [2].

Visual function can be affected in a variety of different ways in BSCR. Central visual acuity is generally preserved to 20/40 or better in at least 62% of the eyes; however, blurred vision is reported in more than 80%, nyctalopia in 17.5%, and dyschromatopsia in 8.7% of the cases [2]. Contrast sensitivity changes were found in 92% of the 63 patients (126 eyes) in a single center, cross-sectional study and were related to poor central visual acuity [12]. Various visual field abnormalities, including visual field constriction, generalized diminished sensitivity, and enlarged blind spot, have also been reported [2, 13]. More advanced central visual field testing by microperimetry also showed decreased retinal sensitivity even in patients with inactive disease [14], suggesting that, despite the clinical impression of remission, retinal sensitivity is still affected and does not recover completely. This discrepancy between clinical impressions based on subjective symptoms and imaging methods on one hand and deficits in visual function support the need for the use of objective methods to evaluate vision function status and inform treatment choices and decisions.

It is generally believed that visual electrophysiological testing could detect early changes and could be used to track the disease progression in uveitis and other retinal inflammatory diseases [15, 16]. However, a more comprehensive look at the value and findings of such tests in BSCR is still lacking. This review is focused on the use of visual electrophysiological tests, like electroretinography (ERG), electrooculography (EOG), visual evoked potentials (VEP), and so forth, in BSCR. As objective and quantifiable testing methods, these tests have the potential to be used as measures to track the progression of the BRCS and estimate the efficacy of current or prospective therapeutic approaches.

## 2. Materials and Methods

The literature search included the online sources PubMed, Web of Science, and Embase from 1980 to February 2015. Only references published in English were included. Case reports were excluded.

## 3. Results and Discussion

### 3.1. Electroretinography

The electroretinogram (ERG) is a recording of the bioelectrical response of the eye from a brief light stimulation. The three types most used in the clinic are full-field ERG, pattern ERG (PERG), and multifocal ERG (mfERG).

#### 3.1.1. Full-Field ERG

**Figure 1:**
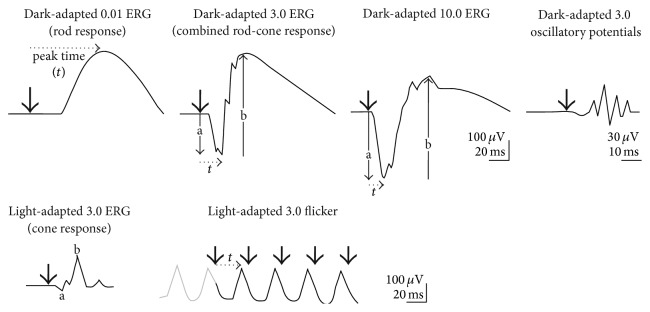
Diagram of the six basic ERGs defined by the ISCEV Standard. These waveforms are exemplary only and are not intended to indicate minimum, maximum, or typical values. Bold arrowheads indicate the stimulus flash; solid arrows illustrate a-wave and b-wave amplitudes; dotted arrows exemplify how to measure time to peak (*t*, implicit time or peak time) (*to be reprinted with permission from* McCulloch et al. ISCEV Standard for full-field clinical electroretinography (2015 update) [17].* Documenta Ophthalmologica: Advances in Ophthalmology*. 2015, 130(1): 1–12.)

The full-field ERG is a retinal response to a diffuse brief (typically less than 5 milliseconds) illumination of the whole retina. As an objective measure of the retinal function, it is well suited not only for tracking the effect of retinal disease progression on the visual function, but also for estimating the efficacy and safety of therapies either at drug development stage or in postmarketing studies [18]. For clinical purposes, this test is most often recorded in compliance with an international standard for full-field ERG recording established by the International Society for Clinical Electrophysiology of Vision (ISCEV), first published in 1989 and regularly updated after that [17, 19]. The current edition of the ISCEV Standard recommends as a protocol the collection of four dark-adapted and two light-adapted types of responses and prescribes how the responses are recorded, measured, analyzed, and reported (Figure 1).

At least 21 peer-reviewed articles (Table 1) have been published using some parameters of the full-field ERG to support diagnostic or therapeutic decisions in the management of BSCR since 1980. Some of the early works [8, 20–22] provide very little detail about the ERG methodology and that makes it difficult to compare their findings with more recent studies. Although a variety of ERG parameters have been reported to be affected in different ways, from supernormal responses at the early stages to greatly diminished and delayed or even absent responses at late stages, an informal consensus has emerged about 10 years ago that the light-adapted 30 Hz flicker response is the most sensitive one [23–25] and it has since then been used as an outcome measure in clinical trials testing drug effectiveness in this condition [26–28]. Therefore, the following analysis of studies reporting full-field ERG results will be centered on reported changes in this response.

The first better documented study in terms of ERG methodology detailed by Fuerst et al. [29] demonstrated for the first time that ERG 30 Hz flicker peak time is prolonged significantly and more than any other peak time ERG parameter in all six patients participating intuit study. Few years later, Hirose et al. [30] studied 15 patients (28 eyes) and concluded that several parameters: the scotopic b-wave, the photopic b-wave, and the 30 Hz flicker response, were all affected in at least 50% of the eyes. They did not measure (or at least report) 30 Hz flicker peak time. It is worth pointing out that all of the above-mentioned works, as well as some more recent ones [31–33], did not use a methodology fully compliant with the ISCEV Standard. That makes it very difficult to compare directly results between different studies and to make conclusions about comparative diagnostic and/or management utility of the different ERG parameters used.

In 2002, the first work evaluating full-field ERG as an indicator of disease activity and using a recording protocol conforming to the ISCEV Standard was published by Zacks et al. [23]. The authors compared seven parameters of the ERG responses from 15 HLA-A29-positive BSCR patients (30 eyes) at age 29–69, who were on immunosuppressive medication and had three serial ERG recordings. They correlated the ERG parameters with the success in tapering immunosuppressive therapy and concluded that the ERG parameter that showed the most significant correlation with an inability to taper medication was the light-adapted 30 Hz flicker implicit time (*p* < 0.01). The only other parameter that reached statistical significance in that comparison was the combined rod-cone ERG b-wave amplitude (*p* < 0.05). Given the relatively small number of patients involved, the fact that two out of the seven ERG parameters reached statistical significance was an indication of the potential of this test to be used in monitoring the therapeutic effectiveness in BSCR.

More support for the idea that the 30 Hz ERG flicker response may be useful in BSCR came in 2005 with the work of Holder et al. [24], who used a variety of ERG parameters (standard and some nonstandard) in a cohort of 18 patients with BSCR (age: 26–64 years). Nine out of 10 patients who had systemic treatment with steroids and/or immunosuppressant drugs and serial ERG recordings showed improvement in some ERG parameters. The authors concluded that the most sensitive ERG parameter was the light-adapted 30 Hz flicker implicit time in direct confirmation to the previous findings of Zacks et al.

The only other detailed study from the same period with a main goal to evaluate the use of ERG in monitoring BSCR was that of Sobrin et al. [25], who studied 23 BSCR patients (age range: 39–75) over the course of several years (up to 7 years). Although the authors concluded in their Discussion that “the best parameter to detect improvement during treatment is not clear,” their data show a very significant effect on light-adapted 30 Hz flicker peak time as a delayed response (*p* < 0.001) at baseline and during observed intervals without treatment (*p* = 0.001). Only 9 patients (18 eyes) out of the 23 had more than one ERG follow-up during treatment; in this subset, the ERG parameter that showed the most significant change was the 30 Hz flicker amplitude (*p* = 0.002), while the 30 Hz flicker peak time did not show significant change (*p* = 0.13). The lack of change in 30 Hz flicker peak time may be due to the insufficient number of subjects, short time of observation, and so forth.

Since then, two other studies showed also delay in 30 Hz flicker peak time in BSCR patients. Kiss et al. studied 28 patients, who were followed up every 6 months with ERG [34]. They reported that 58% of the patients had delayed 30 Hz flicker peak time at the initial visit and 62.5% at the final visit. Sobrin et al. did retrospective analysis of the ERG records in 8 patients (16 eyes) treated with intravenous application of daclizumab at 2-week intervals for 25-month period [35]. They found that the ERG 30 Hz peak times were further delayed by 2 milliseconds or more at the last available ERG for each patient in 4 eyes, including two eyes of two patients with adequate inflammation control.

Furthermore, four studies used this parameter in monitoring the therapeutic efficacy in BSCR, either as part of the clinical definition of relapse/inflammation control (infliximab therapy) [27], as part of the overall management decision process [26, 28] (fluocinolone, cyclosporine A, and mycophenolate), or as a secondary outcome measure [36] (mycophenolate). However, only two studies [27, 36] reported statistical results indicating that 30 Hz flicker peak times were significantly shorter at the end of the follow-up period, in parallel with improvement in angiographic and clinical signs.

Another aspect of the full-field ERG changes in BSCR is the dependence of the ERG changes on the stage of the disease, for example, in early versus late disease. This aspect has not been explored in detail and only very few studies have adequate information covering this relationship, as the natural course of the disease is uncertain. One such work is the study by Hirose et al. [30] based on the results of 15 patients (28 eyes) where the authors reported the effects of duration of disease on ERG parameters. Generally, they find decline in the a-wave and b-wave amplitudes with progression of the disease, without providing a correlation coefficient. They estimate two groups of patients: one with acute decline in retinal function and one with more gradual decline in retinal function. Thus, it is likely that ERG amplitudes reflect the state of the retina related to the stage of the disease.

#### 3.1.2. Pattern ERG

Pattern ERG (PERG) reflects ganglion cell activity in the central retina. Thus, similar to visual field changes in BRCS as described above, PERG would be expected to demonstrate negative changes (decrease in amplitude or delay in peak time) reflecting the current functional status of the macula. Very few studies have been published using PERG in BSCR. Thus, Holder et al. recorded pattern ERG in 18 patients in parallel with full-field ERG [24]. In most patients, PERG changes were similar to the full-field ERG changes in terms of amplitude decrease and peak time delay and were correlated with color contrast sensitivity changes.

#### 3.1.3. Multifocal ERG

The multifocal ERG (mfERG) represents an array of local, cone-driven responses from the central 20 to 30 degrees of the retina [37]. The main contribution of the mfERG signal comes from bipolar cells, with smaller contributions from amacrine or ganglion cells [38, 39]. Only two reports have been published to date using mfERG in BRCS. Birch et al. [40] reported that 6 eyes with anatomical thinning in the macula, both in terms of reduction in total retinal thickness and outer retinal thickness and defined by SD-OCT, had significantly lower mfERG responses compared to 8 eyes without anatomical thinning and all eyes with a history of BRCS for more than 10 years had abnormal mfERG response densities. In a more recent study, Chiquet et al. reported significant decrease in amplitudes and prolongation of peak times in 28 patients with BRCS and these changes were well correlated with visual acuity, mean defect of visual field, foveal threshold, and color vision score and with fluorescein angiography results [41]. These two studies clearly demonstrate the potential of mfERG as an objective test of the central retinal function in BR, although more comprehensive studies would be clearly beneficial, especially comparing how the mfERG changes reflecting macular function compare with extramacular changes, which would be better reflected in full-field ERG.

### 3.2. Electrooculography

Electrooculography is a technique for measuring the standing potentials of the eye, generated mostly in the retinal pigment epithelium (RPE) cells [42]. As the choroidal lesions, which represent a hallmark of the disease, are located directly below the RPE, it is expected that EOG would be affected. Indeed, in a review article about BRCS, Shah et al. summarized the results of six studies applying EOG including a total of 170 eyes and estimated that 66% of the tests had abnormal Arden ratios, the main EOG parameter [2]. However, EOG has not been used as a test to follow BRCS progression since the mid-80s, probably because of low sensitivity and variability of the responses.

### 3.3. Visual Evoked Potential

Visual evoked potentials (VEP) are visually evoked signals extracted from the EEG activity in the visual cortex recorded from the overlying scalp, most often as a result of a flash or pattern stimulation. As flash VEP amplitude shows a large degree of interindividual variability, pattern VEP recorded typically to central 15 degree reversing monochrome pattern stimulus is currently preferred for clinical standardized testing [43]. Priem and Oosterhuis [7] reported abnormal flash VEP amplitudes in 53% and increased latency in 22% of 30 patients (60 eyes) tested with BSCR. Priem et al. [21] reported affected pattern VEP responses as reduced amplitudes and prolonged peak times in most of the 16 patients (32 eyes) that they tested. In the same patients, they found normal flash VEP responses. No studies have been published after the introduction of the first edition of the International standard for VEP recording [44] and, thus, it is presently unclear whether standardized recordings may improve the sensitivity or specificity of the VEP responses.

## 4. Discussion

Although many studies have been conducted over the years, there is still some uncertainty and ambiguity regarding the best electrophysiological method (or a specific parameter within a method) to quantify changes in retinal function associated with the course of BRCS. One of the reasons for that is the scarcity of experimental models of BRCS. Another reason is that some ERG responses are smaller and difficult to record in mice, the most widely used species for animal models of posterior uveitis. This is the case with the photopic 30 Hz flicker response and, to the best of our knowledge, no studies have been published demonstrating changes in this response in mouse models of uveitis. However, other ERG parameters have been successfully used in animal models and have been shown to be an effective objective indicator of retinal function and in monitoring the disease progression and therapeutic safety and efficacy [45–47].

The cellular mechanism responsible for the high sensitivity of 30 Hz flicker ERG response to retinal changes caused by BRCS is currently unknown. Several hypotheses can be put forward to explain the observed relatively strong correlation between clinical disease progression and this type of ERG response. One such hypothesis would place emphasis on the fact that, for the retina to be able to follow precisely the relatively fast 30 Hz flicker stimulation, its metabolic and physiological pathways have to operate at higher rate and any disturbance in it would result in degraded performance. Such a hypothesis is supported indirectly by the documented decline in flicker fusion frequency observed electrophysiologically in experimental posterior uveitis [48], although studies in BRCS are lacking. Similar sensitivity of the 30 Hz flicker ERG response to progression of a vascular retinal disease is reported in central retinal vein occlusion (CRVO) for dark- [49, 50] and light-adapted flicker response [51, 52] and also in branch retinal vein occlusion (BRVO) [53].

Of note, although the highest concentration (density) of cone photoreceptors and cone bipolar cells is in the fovea, the overall number of cone photoreceptors in the fovea is only about 200,000 or ~4% of the total number of cone photoreceptors in the retina [54, 55]. As the 30 Hz flicker response is generated predominantly by cone bipolar cells [56–58], it should not be surprising that this parameter is sensitive to a disease that demonstrated pathological changes mostly in retinal periphery. On the other hand, this can explain the lack of correlation between ERG parameters and central visual field (Humphrey Visual Field 24-2) changes in a recent longitudinal study in BSCR [59]. Of note, 30 Hz flicker peak time was also the most affected ERG parameter. On the other hand, the question sometimes arises as why visual function in the macula can be affected as detected by PERG or mfERG, especially in cases without macular edema, while most of the lesions are extramacular. In this context, localized pathological changes in the choroid can have widespread visual function effects, often extending beyond the circumscribed choroidal lesion, as has been demonstrated in experimental photodynamic therapy [60].

The full-field ERG records a mass response reflecting activity throughout the retina and is dominated by signals coming from the retinal periphery. As most of the observable changes in BSCR are also located in the periphery, it would be expected that full-field ERG parameters would be more sensitive to disease progression, compared to electrophysiological tests of more central retinal function, like PERG and standard mfERG, although a detailed comparison between the three methods is not available. Thus, it may be expected that full-field ERG parameters, like the 30 Hz flicker response, will continue to play an important role in the evaluation of retinal function in BSCR in the near future. A more sensitive (and specific?) test may be wide field mfERG, either alone or in combination with full-field ERG [61].

In summary, there is large clinical evidence and precedent (based on results from more than 200 patients) to support the use of light-adapted 30 Hz flicker ERG response as an outcome measure in clinical trials involving BSCR and in clinical practice. When recording this response, both amplitude and implicit time are parameters that are available and it may be beneficial to record and analyze both parameters.

However, it has to be kept in mind that some large studies with a long follow-up period have been inconclusive [25] and the majority of the 30 Hz ERG results are reported from only a few centers. This is understandable, as BSCR is a relatively rare disease and recruitment of a sizeable number of patients can be a challenge by a single center. Another limitation is that the direct effect of immunosuppressive therapy, often used in BSCR, on the ERG signal (including 30 Hz flicker) has not been reported. As systematic studies are lacking, some caution should be applied and this should be investigated in future studies.

At present, a well-designed multicenter study to systematically evaluate and compare different electrophysiological methods or parameters in BSCR bases on sensitivity and specificity is still lacking. Such a study could be very useful in providing an objective assessment of retinal function during the course of the disease and help evaluating the overall treatment efficacy and the dosing regimen of newly developed or established pharmaceutical agents.

## 5. Conclusions

Various visual electrophysiology tests have been used over the years to document various vision function changes in BRCS and many of them show different degree of abnormality. Historically, the photopic 30 Hz flicker peak time of the full-field ERG has emerged as the most popular parameter to follow the disease progression and estimate the success of treatment. The exact mechanism behind the observed 30 Hz peak time delay in BRCS is unclear. It also remains uncertain whether this is truly the mist sensitive parameter, which can be only established with a large, longitudinal, multicenter study to evaluate the value of different electrophysiological methods or parameters. Until then, there is enough evidence to recommend the use of photopic 30 Hz flicker in the clinical management of BSCR.

## Figures and Tables

**Table 1:** Studies reporting full-field ERG results in birdshot chorioretinopathy.

Authors	Year	Type of study	Patients *n*/ERG *n*	Eyes *n*/ERG *n*	Disease severity	ISCEV Standard	Rod ERG amplitude	Rod ERG peak time	Mixed ERG amplitude	Mixed ERG peak time	Photopic b-wave amplitude	Photopic b-wave peak time	Flicker ERG amplitude	Flicker ERG peak time	Note 1	Note 2
Kaplan and Aaberg [20]	1980	Case reports	4	8	Low/ variable				↓	→	N	N			Method is unclear	

Gass [8]	1981	Cross-sectional	11/10	22/20	Variable										No detailed breakdown of ERG changes by stimulus/parameters	Abnormal rod and cone ERGs in all 10 patients

Fuerst et al. [29]	1984	Cross-sectional	9/6	18/10	Variable		↓↓	→			↓	→	↓	→ →	Good ERG method	

Priem et al. [21]	1988	Cross-sectional	16	32 ?	Variable										Poor ERG method, few details	b-wave/a-wave ratio correlated with vasculopathy

Godel et al. [22]	1989	Case reports	2	4	Severe/ moderate		↓		↓	→					Few ERG method details	ERG got worse with disease progression

Hirose et al. [30]	1991	Cross-sectional	15	28	Variable				↓		↓↓↓		↓↓		Good ERG method, each ERG component affected to a different degree	

Fich and Rosenberg [31]	1992	Case reports	2	4	Severe				↓↓	→ →			↓↓	→ →	Few ERG details	

Gasch et al. [32]	1999	Cross-sectional	59/22	??/44 ?	Variable		↓		↓		↓		↓		No detailed breakdown of ERG changes by stimulus/parameters	Equal reduction in rod and cone responses claimed

Oh et al. [33]	2002	Cross-sectional	19/14	??/28 ?	Variable	?	↓↓		↓↓		↓				ERG 30 Hz flicker not done	b-wave/a-wave ratio ↓ initially, then overall decrease

Zacks et al. [23]	2002	Cross-sectional and follow-up	15	30	Variable	Yes	↓		↓		↓		↓	→ →	Correlated 7 ERG parameters with ability to taper immunosuppressive Rx	30 Hz flicker time was the best

Holder et al. [24]	2005	Cross-sectional and follow-up	18	36	Variable	Yes									30 Hz flicker amplitude and time most sensitive	ERG classified only as normal/abnormal

Sobrin et al. [25]	2005	Cross-sectional and follow-up	23	46	Variable	Yes	↓		↓		↓		↓	→ →	The authors state that most sensitive parameters are unclear	Tables 2, 5 and Figure 4 suggest 30 Hz flicker; time most affected

Kiss et al. [34]	2005	Cross-sectional and follow-up	28	56	Variable	Yes			↓					→ →	Only 2 ERG parameters monitored: mixed amplitude and flicker time	

Sobrin et al. [35]	2008	Cross-sectional and follow-up with Rx	8	16	Variable	Yes	↓		↓		↓			→ →	Reported numeric values for only 2 ERG parameters: 30 Hz time and max amplitude	Too few patients for statistics

Thorne et al. [13]	2008	Cross-sectional and follow-up	55/24	109/48	Variable	?									No detailed breakdown of ERG changes by stimulus/parameters	79% had abnormal ERG

Rush et al. [26]	2011	Rx follow-up	19	32	Variable	Yes							↓	→ →	Used flicker ERG for follow-up, amplitude, and PT	Only ERG flicker reported; no statistics

Artornsombudh et al. [27]	2013	Rx follow-up	22	44	Variable	Yes									Used flicker ERG time for follow-up as part of the definition of relapse/inflammation control	No ERG statistics

Cervantes-Castañeda et al. [28]	2013	Rx follow-up	49	98	Variable	Yes									Used flicker ERG amplitude and time for follow-up	Time is more sensitive than amplitude

Doycheva et al. [36]	2014	Rx follow-up	24/21	48/42	Variable	Yes									Used mixed flicker ERG for follow-up	No change in ERG parameters; effective Rx?

Rx: treatment.
